# Effect of the Different Growth Shapes on the Electrochemical Behavior of Ti Thin Films for Medical Applications

**DOI:** 10.3390/ma18173959

**Published:** 2025-08-24

**Authors:** Matteo Bertapelle, Joel Borges, Julia Claudia Mirza-Rosca, Filipe Vaz

**Affiliations:** 1 Mechanical Engineering Department, University of Las Palmas de Gran Canaria, 35001 Las Palmas de Gran Canaria, Spain; 2 Physics Centre of Minho and Porto Universities (CF-UM-UP), University of Minho, 4710-057 Braga, Portugal; 3 LaPMET—Laboratory of Physics for Materials and Emergent Technologies, University of Minho, 4710-057 Braga, Portugal; 4 Materials Engineering and Welding Department, Transylvania University of Brasov, 500036 Brasov, Romania

**Keywords:** thin film, titanium, medical applications, SEM, EIS, growth geometry

## Abstract

The response of titanium (Ti) thin films is closely related to their microstructure, which is extremely sensitive to the selected deposition parameters and geometrical configurations. The present study investigates the impact of geometrical factors on the growth of Ti thin films, focusing on how variations in growth geometry influence film microstructure, surface morphology, and corrosion resistance. Three Ti thin films were prepared using Glancing Angle Deposition (GLAD) in a custom-built DC reactive magnetron sputtering system. For the first sample, the target was positioned perpendicular to the substrate surface (α = 0°); for the second and third samples, the substrate holder was positioned at an angle of 85° regarding the target direction (α = 85°), incorporating a 180° azimuthal rotation for the last (to obtain a zigzag-like deposition). The thickness and morphological features of the thin films were investigated by SEM, while the surface morphology, specifically roughness, and crystallinity of the thin films were assessed by AFM and XRD, respectively. Continuous and alternating current techniques were used for electrochemical characterization of behavior in simulated body fluid. The obtained results show a clear tendency to an improvement in anticorrosion performances varying the nanoarchitecture of the films in comparison to the conventional-grown sample, with the inclined sample presenting a slight enhancement in corrosion resistance and the zigzag-grown sample having the best corrosion resistance properties of the three.

## 1. Introduction

Titanium (Ti), one of the most studied transition metals, has attracted considerable attention in thin film research due to its outstanding mechanical strength, excellent corrosion resistance, and notable biocompatibility [1,2,3]. These properties make this material highly suitable for a wide range of advanced technological applications, including microelectronics, biomedical implants, optical coatings, and aerospace components [4,5,6]. In its thin film form, Ti also exhibits distinctive physical characteristics such as tunable electrical resistivity, strong adhesion to various substrates, and the capability for precise nanostructuring, rendering it especially advantageous for functional coatings and emerging nanotechnologies [7,8,9,10,11,12,13,14].

In terms of its behavior, the response of Ti thin films is closely related to its microstructure, which is extremely sensitive to the selected deposition parameters and geometrical configurations [7,8,9,10,11,12,13,14]. Factors such as substrate orientation, deposition angle, and surface topography critically influence the resulting thin film morphology and crystallographic texture [1,2,3,4,5,6,15]. These geometrical variables affect key processes during film growth, including nucleation, adatom diffusion, columnar grain evolution, and atomic shadowing effects [5,15,16,17,18,19,20].

Among the different reported techniques for its thin film-like synthesis, Glancing Angle Deposition (GLAD), where the vapor flux impinges on the substrate at an off-normal angle, has been shown to induce important anisotropic microstructures, characterized by tilted columns, increased porosity, and self-organized nanostructures, just to mention a few [15,21,22]. Such unusual morphologies are particularly beneficial for applications requiring low-density structures, high surface area, or directional properties, including sensors, catalysts, biomedical (especially in surfaces for cell cultures), and photonic devices [15,21,23,24]. In contrast, deposition at normal incidence generally yields denser films with equiaxed grains, which are preferred for barrier coatings and electronic applications [24]. Additionally, substrate geometry, whether flat, curved, patterned, or structured, introduces further complexity to the growth dynamics. For example, patterned substrates can create local variations in growth rates and shadowing, resulting in hierarchical or nanostructured surfaces [25]. These structural modifications can significantly alter the film’s chemical, mechanical, optical, and tribological properties, presenting opportunities to engineer surface functionality for specific applications [1,2,3,4,5,6,7,8,9,10,11,12,13,14].

Beyond these important fields, which one finds in the frontline for most common applications, it is also widely known that engineering materials used in industry need to consider their chemical resistance, namely that related to corrosion, as the degradation of materials through corrosion has an excessive impact on the economy, human life, and the environment [26]. The most common kinds of corrosion result from electrochemical reactions occurring when most or all the atoms on the same material surface are oxidized, damaging the entire surface. Most materials are easily oxidized, tending to lose electrons to oxygen (and other substances) in the air or in water. As oxygen is reduced (gains electrons), the corroded material forms an oxide with the metal [27,28,29,30].

In the case of biomaterials research (such as the present work), corrosion plays a crucial role in the performance and application of thin films, influencing their durability, biocompatibility, and functionality in medical and engineering contexts. While corrosion is often perceived as a degradation process to be minimized, in biomaterials, controlled corrosion can be essential for specific applications, such as biodegradable implants, drug delivery systems, and tissue engineering scaffolds [31,32]. Many biomedical implants, such as stents and orthopedic fixtures, are designed to degrade over time within the body. Any thin film system for such biomedical applications relies on controlled corrosion to dissolve gradually, providing temporary mechanical support while avoiding the need for secondary removal surgeries. Understanding corrosion mechanisms helps tailor degradation rates to match tissue healing processes [31]. Furthermore, corrosion can enhance the bioactivity of biomaterial surfaces by forming oxide layers or releasing ions that promote tissue integration. For example, Ti thin films develop a passive oxide layer (TiO_2_) that improves corrosion resistance while maintaining biocompatibility. In contrast, controlled corrosion of bioactive glasses can release calcium and phosphate ions, stimulating bone regeneration [31,32].

Respecting this, GLAD thin films play a crucial role in biomedical implants by enabling the fabrication of highly controlled and complex nanostructures on implant surfaces. These nanostructures significantly enhance biocompatibility, promote bone integration (osseointegration), and can impart antibacterial properties. By precisely manipulating the deposition angle and substrate motion during the GLAD process, it is possible to tailor the surface architecture at the nanoscale. This level of control allows for the design of surfaces that actively influence cell behavior and tissue response, ultimately improving implant performance and longevity.

Taking this into account, the present study investigates the impact of geometrical factors on the growth of Ti thin films, focusing on how variations in growth geometry influence film microstructure, surface morphology, and corrosion resistance. Through a systematic exploration of these relationships, this work aims to elucidate the underlying mechanisms that govern film behavior and to identify strategies for tailoring Ti coatings for targeted applications, which in this case are the potential use of these films for human implant materials protection. Continuous and alternating current techniques were used for electrochemical characterization of behavior in simulated body fluid of the different prepared microarchitectures. Using these techniques, it was possible to evaluate the stability of the corrosion layers that were formed and the contribution of eventual resistance or capacitance elements, thanks also to the equivalent circuit fitting.

## 2. Materials and Methods

### 2.1. Thin Film Deposition

**Figure 1:**
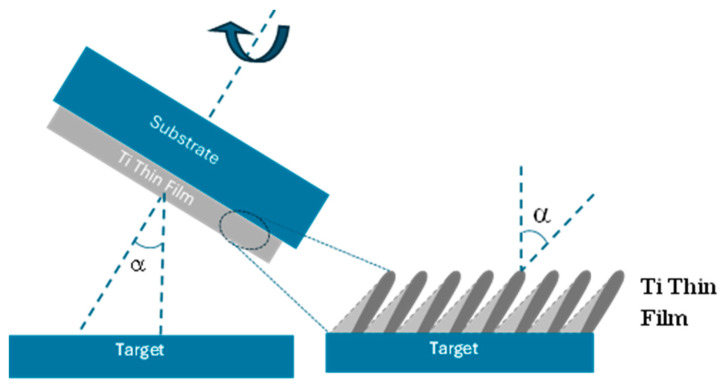
Schematics of the deposition system used for the preparation of the three Ti thin films.

Three titanium (Ti) thin films were fabricated using GLAD in a custom-built DC reactive magnetron sputtering system [33]. In GLAD, the substrate is positioned at a shallow angle relative to the incoming flux of the target material, creating an oblique deposition geometry (Figure 1). This setup promotes anisotropic film growth across the substrate surface. During film formation, incoming atoms condense and nucleate spontaneously. Due to ballistic, line-of-sight shadowing effects, these nuclei obstruct the flux, preventing it from reaching the areas behind them. Consequently, tilted columnar structures develop, aligned with the direction of the incoming material flux.

The first Ti sample was deposited using the conventional thin films deposition geometry configuration, in which the target is positioned perpendicular to the substrate surface (α = 0°).

Two additional samples were prepared by modifying the orientation of the substrate holder, specifically by adjusting the geometrical angle between the target and the substrate to α = 85°. This configuration enabled the deposition of a thin film under conditions hereafter referred to as the inclined sample. Furthermore, by incorporating a 180° azimuthal rotation, a zigzag deposition geometry was achieved; this configuration is referred to in this work as the zigzag sample. Additional schematic details and further information about the GLAD deposition (Glancing Angle Deposition) geometry are available elsewhere, in another publication of the group [33].

During deposition, the target was sputtered using a current density of 75 A/m^2^ in an argon atmosphere (Ar flow rate: 25 sccm; partial pressure: 3.5 × 10^−1^ Pa). The base pressure in the deposition chamber was maintained below 5 × 10^−4^ Pa for all three samples. Prior to deposition, substrates were cleaned with ethanol to eliminate surface molecular contaminants. To enhance surface energy and improve film adhesion, plasma activation was performed using a low-pressure Plasma Cleaner (Diener Electronic Zepto Model, Ebhausen, Germany) equipped with a 13.56 MHz RF generator [34]. The activation process was conducted in an Ar atmosphere (0.80 mbar) for 15 min with an applied power of 50 W.

Deposition time was adjusted to ensure that all samples reached a similar film thickness of approximately 1.0 ± 0.1 μm. Both glass and silicon substrates (boron-doped, p-type, monocrystalline, 100 orientation) were employed, mounted on a grounded hexagonal substrate holder placed 70 mm from a high-purity Ti target (99.99%, dimensions: 200 × 100 × 6 mm^3^).

### 2.2. Structural and Morphological Characterization

The thickness and morphological features of the thin films were characterized by Scanning Electron Microscopy (SEM), using a high-resolution FEI Nova NanoSEM 200 microscope (Thermo Fisher Scientific, Waltham, MA, USA). Surface morphology, specifically roughness and porosity, was investigated via Atomic Force Microscopy (AFM), employing a high-resolution Nano-Observer AFM microscope (Concept Scientific Instruments, Les Ulis, France) operated in resonant mode. Scans were conducted over areas of 10 × 10 μm^2^ on each sample, at a resolution of 1024 × 1024 pixels and a scan speed of 1 line/s.

The crystallinity of the thin films was assessed through X-ray Diffraction (XRD), using a Bruker D8 Focus diffractometer (Bruker, Billerica, MA, USA), equipped with a copper X-ray tube (Cu-Kα_1_, λ = 1.5406 Å). Measurements were performed in a θ–2θ configuration, using a step size of 0.02° with a counting time of 0.2 s per step, over a 2θ range from 20° to 80°.

### 2.3. Electrochemical Characterization

The corrosion behavior of the three thin films, prepared with varying growth geometries by GLAD, was investigated using the EC-Lab^®^ v9.55 software and a Biologic SP-150 potentiostat (Bio-Logic Science Instruments SAS, Seyssinet-Pariset, France), in a simulated body fluid (Ringer’s solution). The software allowed data plotting, calculation of polarization resistance (Rp), determination of Tafel slopes, and extraction of additional electrochemical parameters.

All experiments were conducted in a flat-faced electrochemical cell, specially designed for these types of samples (see attached figure), leaving an exposed material surface of 1 cm^2^. The sample is sealed to the cell wall with a Teflon gasket. To avoid the crevice corrosion process that can occur at the sample/seal interface, an inert material, in our case Teflon, is used for the seal that fits the sample well and is tightly pressed, without leaving spaces for liquid accumulation, thus avoiding the appearance of crevices. A saturated calomel electrode (SCE) served as the reference electrode, a platinum electrode as the counter electrode, and the thin film samples acted as the working electrode. Prior to testing, samples were immersed in Ringer’s solution for 1 h to stabilize the open-circuit potential (OCP).

Corrosion tests were repeated three times for each sample to ensure result reproducibility and data reliability. Linear polarization tests were performed to determine the relationship between applied potential and current response at the corrosion potential (Ecorr). These electrochemical measurements were carried out in accordance with ASTM G102-89 [35], using a sweep rate of 1.66 mV/s over a potential range from −0.1 V to +1 V relative to Ecorr. The current flow through the solution was continuously monitored during the tests.

Additionally, Electrochemical Impedance Spectroscopy (EIS) was conducted following ISO 16773-1-4:2016 [36]. Single sine wave perturbations were applied across a frequency range from 10^−2^ to 10^5^ Hz. The resulting impedance spectra were analyzed using an equivalent electrical circuit model to fit the experimental data.

## 3. Results and Discussion

### 3.1. Morphological Characterization

It is well established that thin films prepared by GLAD exhibit unique and highly tunable morphologies that critically influence their performance in various applications. In this deposition geometry, thin film growth is dominated by atomic shadowing effects, a preferential deposition mechanism where incident vapor flux accumulates on protruding surface features while leaving recessed regions underdeveloped. This anisotropic growth process results in the formation of highly porous, columnar nanostructures with tailored architectures.

Since GLAD is typically performed at low substrate temperatures, thermally activated surface diffusion is significantly suppressed. Consequently, the final film morphology is primarily governed by geometric shadowing rather than adatom mobility, enabling precise control over nanostructural characteristics such as porosity, column tilt, and surface roughness. This distinctive growth mechanism allows for the engineering of thin films with customized optical, mechanical, and chemical properties, making GLAD a powerful technique for applications ranging from photonics to biomedical coatings [15].

**Figure 2:**
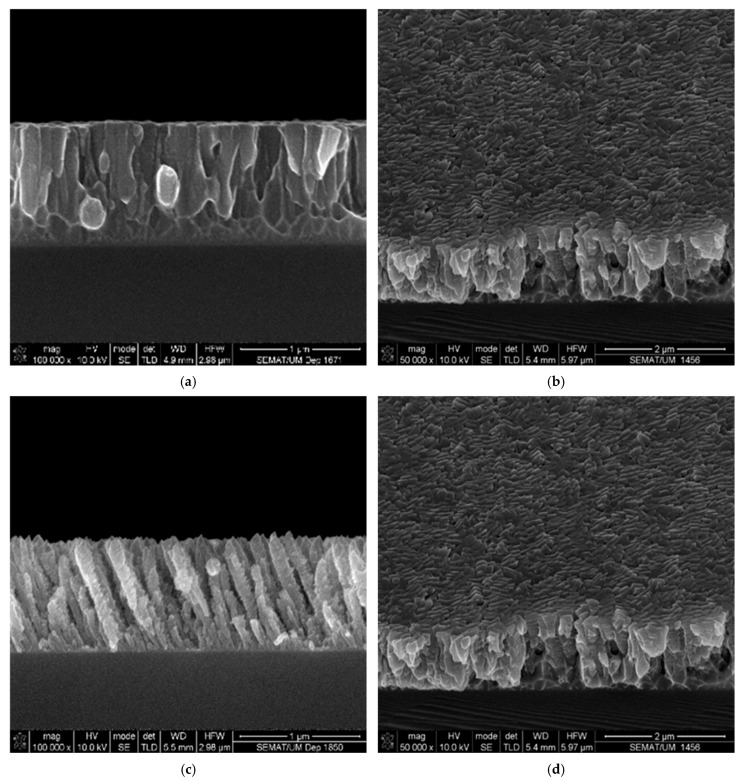

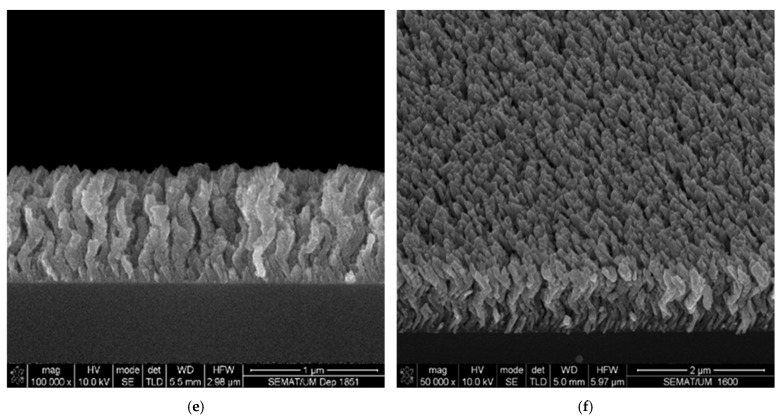
SEM images of the three titanium samples: normal incidence sample cross-section view (**a**) and surface view at tilted angle (**b**); inclined-grown sample cross-section view (**c**) and surface view at tilted angle (**d**); zigzag-grown sample cross-section view (**e**) and surface view at tilted angle (**f**).

Figure 2 presents surface and cross-sectional micrographs of the three fabricated samples, revealing well-defined columnar morphologies. These microstructural features indicate that the films were grown within the transition regime between Zone T and Zone II of the Structural Zone Model (SZM). This classification follows established growth frameworks, including the original SZM proposed by Thornton [37], correlating microstructure with deposition temperature and pressure, subsequent refinements by Petrov et al. [38] and Movchan [39], incorporating additional growth parameters, and the extended model by Mahieu et al. [40,41], which accounts for oblique-angle deposition effects.

**Figure 3:**
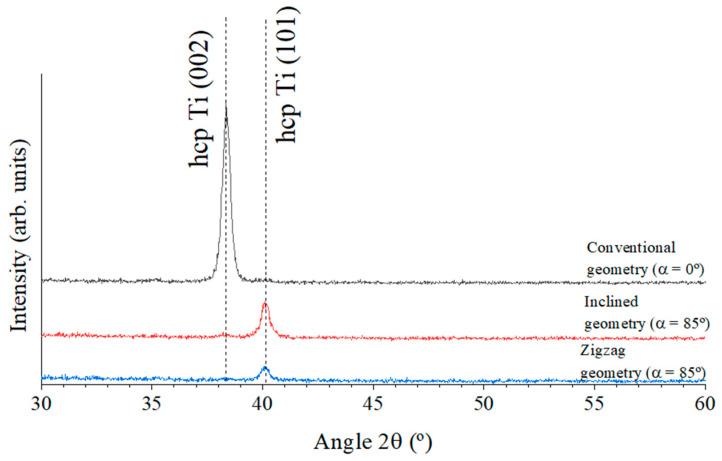
Evolution of the XRD patterns of the Ti thin films prepared at normal incidence, inclined, and zigzag geometries.

Regarding the particular growth features, Figure 2a,b show that the thin film deposited using conventional geometry (α = 0°) exhibited a dense columnar microstructure with well-defined, nearly vertical columns. This morphology is characteristic of thin film growth dominated by surface energy minimization, where adatoms preferentially arrange along crystallographic planes with the lowest surface energy to achieve maximum thermodynamic stability. This type of morphological growth behavior is commonly observed at higher deposition rates, as is the case in this study. The thin film grown under conventional geometry reveals a deposition rate of 24 nm/min, which is approximately three times higher than that of films grown with the inclined and zigzag geometries (~8 nm/min for both samples). Moreover, structural analysis conducted by XRD confirms this growth along the lowest surface energy planes, as evidenced by Figure 3.

As shown in Figure 3, all the Ti thin films crystallized in a hexagonal close-packed (hcp) structure (space group P63/mmc), exhibiting a clear shift in preferential orientation from the (002) to the (101) plane. This transition was accompanied by a significant loss of crystallinity, evidenced by the marked reduction in the intensity of the main diffraction peak in both the inclined and zigzag-grown samples. No other diffraction peaks were observed, namely those related to titanium oxide phases.

The (002) plane in hcp Ti corresponds to the close-packed basal plane and generally possesses the lowest surface energy. It is typically favored under conditions where surface energy dominates, such as high deposition rates, low substrate temperatures (as in this case, where the films were prepared at room temperature), high adatom flux, and limited surface diffusion. These conditions are characteristic of the conventional geometry used for thin film growth.

High deposition rates tend to “lock in” the (002) orientation due to kinetic limitations, as there is insufficient time for adatoms to rearrange. In contrast, lower deposition rates, such as those employed for the inclined and zigzag geometries (three times lower as mentioned above), allow greater surface diffusion, enabling atoms to reorganize into more thermodynamically favorable orientations like (101). This increased atomic mobility can also lead to other energetically advantageous configurations. Therefore, the observed change from (002) to (101) preferential orientation in the inclined and zigzag-grown films underscores the significant impact of deposition conditions on film texture and crystallinity, as illustrated in Figure 3.

In contrast to the high-energy particle deposition and plasma ion impingement characteristic of conventional growth (α = 0°), the use of tilted incidence angles in the inclined and zigzag-grown samples introduces additional factors that must be considered in the development of such microstructures. According to Barranco et al. and Alvarez et al. [42,43], GLAD thin films typically exhibit a tilted columnar structure, where the columns are not isolated but in contact with one another. This is primarily a result of the shadowing effect and the low degree of adatom thermalization. Based on the model proposed by these authors, the films prepared using inclined and zigzag-like geometries develop a γ-type microstructure [42,43], characterized by well-defined and isolated tilted nano-columns, as evidenced in Figure 2c–f. A closer inspection of Figure 2c–f reveals that the inclined and zigzag-grown films present a more porous and less dense morphology. These films feature more clearly defined, tubular-shaped, faceted columns, with a noticeable tendency toward column thinning. This morphological trend aligns with the lower deposition rates observed for these samples, as previously discussed.

**Figure 4:**
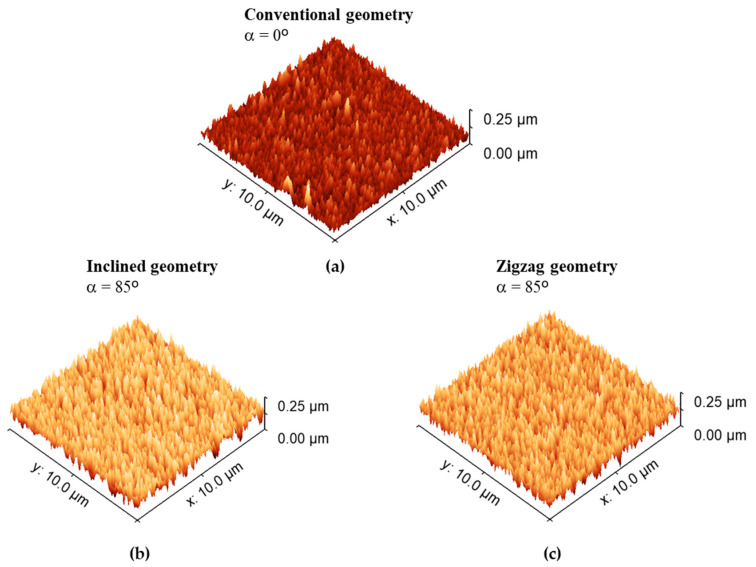
AFM scans of the Ti thin films prepared at normal incidence (**a**), inclined (**b**), and zigzag (**c**) geometries.

Changes in morphological features are also evident from the AFM (Atomic Force Microscopy) analyses performed on all three samples. Figure 4 presents the evolution of surface topography as revealed by AFM. As expected, the different deposition geometries give rise to distinct surface features, in agreement with the SEM observations discussed earlier.

Consistent with previous analyses, Figure 4 reveals two clearly distinguishable surface behaviors among the samples. The thin film deposited under conventional geometry (α = 0°, Figure 4a) exhibits a much smoother surface. In contrast, films grown with inclined and zigzag geometries (Figure 4b,c) display significantly rougher topographies. This behavior is primarily attributed to the reduced penetration depth of the incoming species at tilted angles [43].

At glancing incidence, a greater fraction of the kinetic energy of the impinging atoms is transferred to surface atoms, enhancing adatom mobility. This increased mobility contributes to surface roughening, which may also be partially caused by sputtering effects initiated by oblique-angle bombardment [44]. Consequently, the inclined and zigzag-grown samples exhibit more porous surfaces and less dense microstructures.

This trend is quantitatively supported by the RMS (root mean square) roughness values, which show a notable increase with the change in deposition geometry. While the conventional-grown thin film (α = 0°) exhibits an average surface roughness of approximately 8 ± 0.6 nm, the inclined and zigzag-grown thin films show much higher values, around 32 ± 1.0 nm and 30 ± 1.0 nm, respectively, indicating a fourfold increase in surface roughness.

The set of results presented in Figure 2, Figure 3 and Figure 4 demonstrates that the microstructure and surface characteristics of Ti thin films can be effectively tailored by simply altering the growth geometry. Controlling surface roughness and growth morphology is critically important for a wide range of technical and industrial applications. Such tailoring holds significant relevance in the biomedical field, where surface properties play a key role in the development of materials for human implants.

### 3.2. Corrosion Behavior

**Figure 5:**
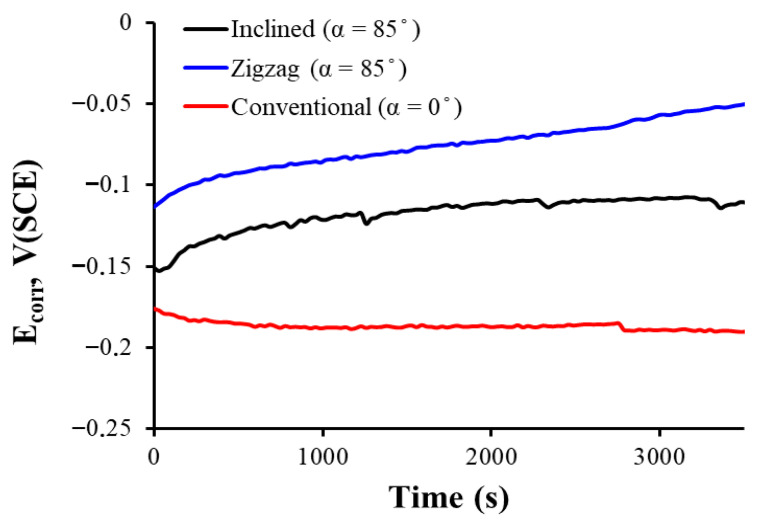
Corrosion potential of the Ti thin films prepared at normal incidence, inclined, and zigzag geometries for 1 h immersion in Ringer solution.

Figure 5 shows how the corrosion potential, E_corr_, of each sample coated with thin film deposited at different angles evolves during one hour of immersion in a medium that simulates the human physiological fluid. In this type of open-circuit potential (OCP) measurement, more positive values are associated with a higher stability of the passive film and, therefore, a lower tendency to corrosion.

Observing the three open-circuit potentials, it is noted that for the sample prepared using conventional geometry (α = 0°), the curve is more negative at the beginning and remains the lowest throughout the immersion, which indicates a lower nobility of the coating and, in principle, a lower protection against corrosion. The two samples prepared with inclined and zigzag-like geometries have higher corrosion potential, which indicates more stable and protective passive films in physiological solution.

**Figure 6:**
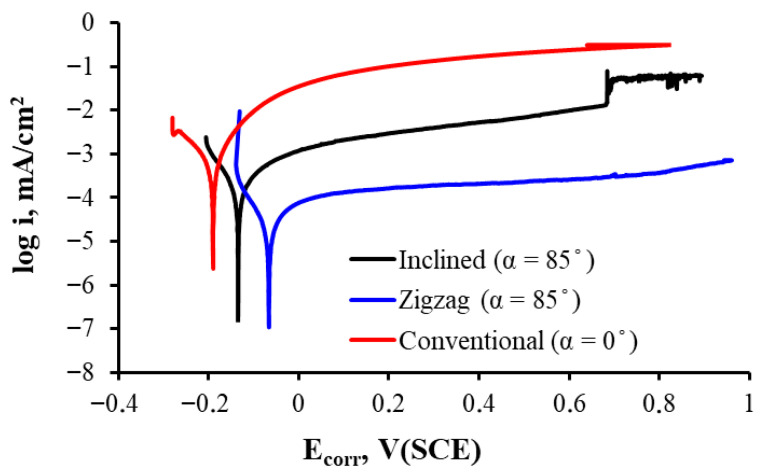
Linear polarization curves of the Ti thin films prepared at normal incidence, inclined, and zigzag geometries in Ringer solution.

The thin film prepared with inclined and zigzag geometries seems to optimize passivation film formation, reaching the noblest corrosion potential and thus showing better corrosion resistance in this simulated physiological fluid immersion test. This can also be observed in Figure 6, where the zigzag-like-grown thin film shows the most positive corrosion potential and the lowest passivation current in all range of potential in the passive zone.

The corresponding corrosion parameters are presented in Table 1. Although all samples have less than 5 at.% oxygen and homogeneous composition, small differences in oxygen distribution or layer compaction could influence the passivation quality. However, since contamination and composition remain fairly constant, the main factor is most likely the columnar structure design and its influence on passivation. Films grown in different geometries exhibit changes in porosity, roughness, and columnar density, as can easily be observed in Figure 2. A steeper growth angle can vary defect density and porosity (which seems rather clear in Figure 2c–f), which influences the formation and stability of the titanium oxide passivation layer.

Titanium is very prone to generating a passive oxide film that gives it good corrosion resistance. However, the rate and effectiveness with which this film forms breaks down and regenerates depend on the topography and microstructure of the surface. For the sample prepared in inclined geometry, the microstructure seems to favor the formation of a more protective film, resulting in a more positive potential. The sample prepared with a zigzag-like design exhibits a nobler corrosion potential, a much lower corrosion current, and a lower corrosion rate, further supported by a very high Rp. All this translates into much higher corrosion resistance for the zigzag-like growth film when compared to that grown in the conventional geometry (α = 0°) and at the same angle in the inclined geometry (α = 85°).

**Figure 7:**
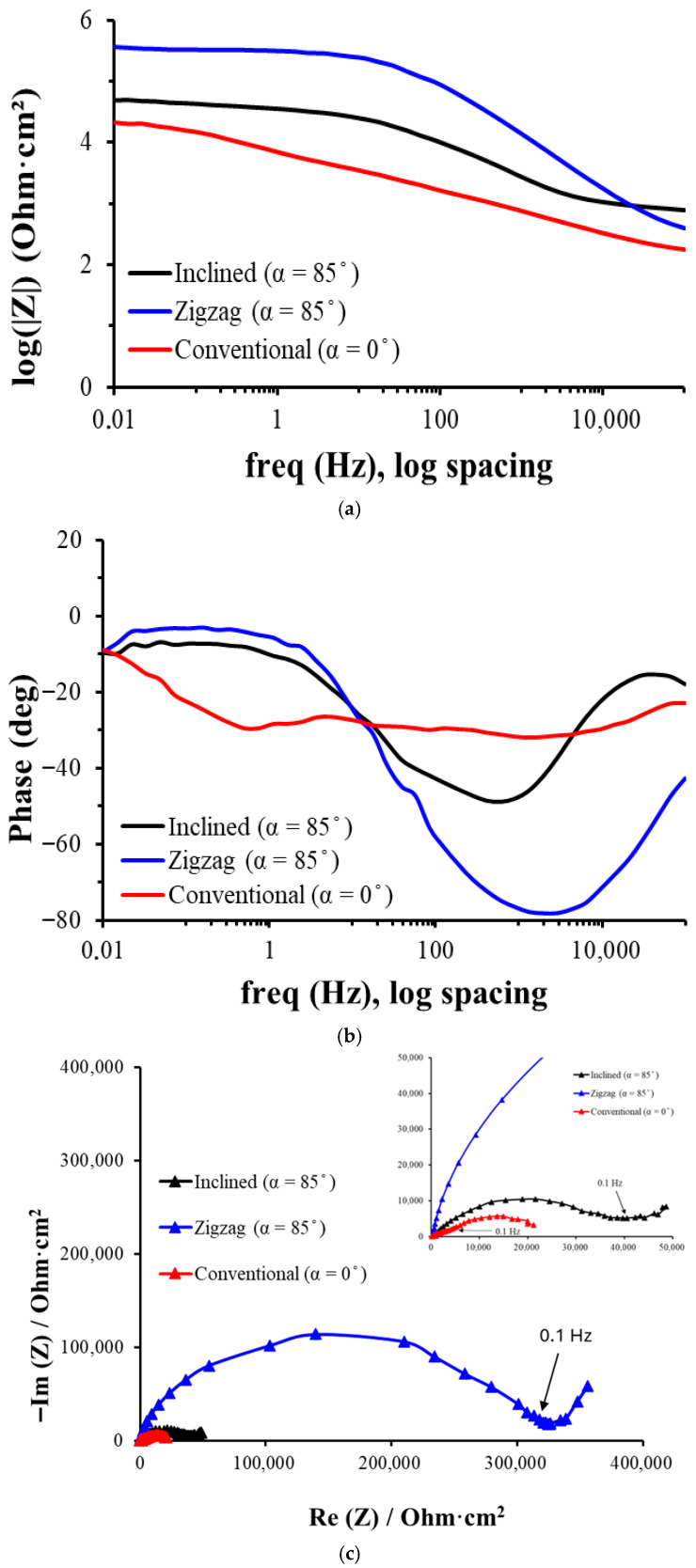
Bode impedance (**a**), Bode phase (**b**) and Nyquist curves (**c**) of the Ti thin films prepared at normal incidence, inclined, and zigzag geometries in Ringer solution.

Figure 7 shows the EIS plots of the three analyzed samples, from which conclusions are drawn on the corrosion resistance, the presence and stability of passive layers, as well as the influence of the microstructure (in this case, the growth geometry of the Ti columns) on the electrochemical behavior. The EIS results are consistent with the measured corrosion potential values; the sample with the more positive potential also tends to show higher corrosion resistance in EIS.

In the high-frequency range (10^4^–10^5^ Hz), all films converge to similar values of IZI, indicating that in this range the ohmic resistance of the electrolyte (R_s_) predominates. Also, the phase values are close to −10° to −20°, indicating that the behavior is predominantly resistive in this range and that there is no significant charge storage at these frequencies.

In the intermediate-frequency range (1–10^3^ Hz), a capacitive dominant region is revealed, associated with the passive layer (oxides, porosity, etc.) when the zigzag film shows the highest impedance, followed by inclined and conventional, suggesting that the zigzag coating offers the highest resistance to charge transfer. Also, the zigzag film reaches phase values close to −75°, indicating a strongly capacitive behavior, typical of a compact or dense passive film, while the conventional layer presents a lower phase (~−45°), suggesting lower barrier efficiency of this layer.

In the low-frequency range (<1 Hz), diffusive and resistive behavior dominates for all three types of samples, relevant for processes such as diffusion of corrosive species and slow dissolution reactions. The zigzag film maintains the highest impedance, indicating better overall corrosion resistance, while the conventional film has the lowest impedance due to a higher permeability, facilitating ion transport. The phase decreases for all coatings, especially in zigzag, which has two distinct minima (~1 Hz and ~2000 Hz), indicating two independent relaxation processes due to two distinct layers. The conventional layer has a smoother and less structured transition, pointing to a more homogeneous but less protective film.

**Figure 8:**
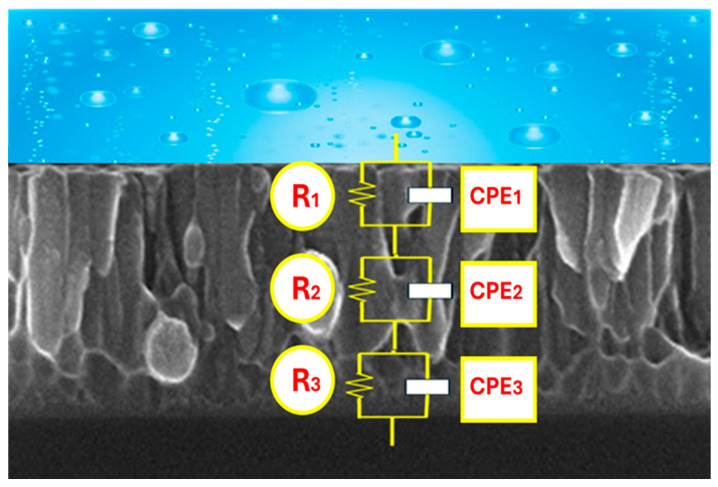
Electrical equivalent circuit used to fit the EIS experimental data of the thin films.

The obtained experimental EIS data have been simulated with an equivalent circuit with three time constants, presented in Figure 8.

Since the experimental data show in the capacitive response dispersion and non-ideal behavior, instead of capacitance, we will use a constant phase element (CPE) whose impedance is represented by the magnitude Y and the exponent n. The CPE, by introducing the exponent n, allows for reflect of this dispersed behavior. When n = 1, there is an ideal behavior, while smaller values reflect the dispersion and deviations of the system. In this way, the CPE is used to obtain a more accurate impedance fit in real systems where ideal models do not fully describe the electrochemical dynamics.

We have used an equivalent circuit model with three R-CPE pairs in series to interpret the electrochemical impedance spectra (EIS) obtained. This choice is based on the hierarchical structure of the coated system, which includes a porous outer layer in contact with the electrolyte, a more compact or dense intermediate layer, which acts as the main barrier against corrosion, and the metallic substrate, where the charge transfer processes take place. The presence of three time constants is also justified by the multiphase response observed in the Bode phase diagrams (see Figure 7b), where three regions with distinct maxima or minima, indicative of independent electrochemical processes in different frequency domains, can be seen. Because in all tests there was the same short distance between the reference electrode and the working electrode, and the Ringer’s solution had a sufficiently high conductivity, the resistance of the electrolyte was equal and very small and was not considered in the circuit.

[R_1_‖CPE_1_] represents the porous outer layer with R1 for electrolyte resistance inside the porous channels and with CPE_1_ for distributed capacitance due to surface roughness and heterogeneity in porosity. [R_2_‖CPE_2_] is associated with the compact interlayer, with R_2_ associated with resistance to load transfer through this dense barrier and CPE_2_ representing non-ideal dielectric behavior of this layer, due to its microstructure. [R_3_‖CPE_3_] represents the charge transfer process at the first deposition of the substrate with R_3_ for polarization resistance of the substrate and CPE_3_ for double layer capacity and final diffusive effects.

The fits performed with this three-time-constant model show excellent agreement with the experimental data. Chi-square (χ^2^) values ≤ 10^−3^ were obtained in all cases, indicating a good quality of fit and confirming the adequacy of the model to the physical system. Moreover, the model reproduces faithfully the number and position of extremes in the phase diagrams and the slope and shape of the log|Z| curve as a function of frequency. These results support both the physical validity of the proposed model and its mathematical robustness to describe the electrochemical processes involved in these thin films on biomaterial.

The experimental results obtained by fitting the experimental data with the equivalent circuit of Figure 8 are presented in Table 2.

The Y parameter is related to the “capacitance”, but considering that it does not behave as an ideal capacitor. A lower value of Y generally suggests a smaller effective area or lower charge storage capacity, which may be due to lower porosity or lower surface roughness.

For the exponent n, values lower than 1 evidence a dispersion of relaxation times, which is associated with heterogeneities at the interface, such as roughness, non-uniform distribution of properties, or variations in composition. The less n deviates from 1, the less the non-ideality or dispersion in the system. The variation observed in the CPE_2_ parameter n2 among the different samples is interpreted as an indication of morphological and structural differences in the compact interlayer, responsible for the passive behavior. An n2 value closer to 1 reflects a more homogeneous and dielectric layer, while lower values of n2 point to heterogeneities or local diffusive effects, probably related to the microstructure generated by the growth geometry. This is not an inconsistency, but an electrochemical manifestation of the different degrees of compaction and quality of the passive layers formed.

For the associated resistance R, a high value indicates a more effective barrier for charge transport or a denser film, while a low value suggests higher conductivity or a more permeable structure.

The parameters derived from the equivalent circuit fit (R, Y, n) allow us to establish clear relationships between the structure of the films formed and their electrochemical behavior. Y1 and n1 (for the outer porous layer) show higher values of Y1 and n1 < 0.9 (for zigzag geometry), indicating a more heterogeneous layer or with higher surface porosity. Conversely, lower values of Y1 and n1 close to 1 (for conventional configuration) suggest a denser or less developed surface. R2 and n2 (for the intermediate compact layer) indicate high R2 and low n2 (~0.5–0.6) (as in zigzag geometry), revealing the presence of an active barrier layer, which imposes limited diffusion and contributes to better long-term passivation.

R3 (for substrate/inner film resistance) in configurations such as zigzag is higher, which indicates greater protection against corrosion due to the thicker and more ordered stacking of the inner layers.

The increase in Ecorr in zigzag suggests a higher tendency to spontaneous passivation, while Rp, which combines all resistances in series, is significantly higher in this geometry, indicating a lower effective corrosion rate.

While structures such as zigzag or inclined show higher corrosion resistance (↑ Rp, ↑ R2) and more effective passive layers (low n2, high R3), possible mechanical compromises must also be considered. First, increased surface porosity and roughness may weaken the mechanical adhesion of the film or generate stress accumulation zones, causing delamination. Second, thicker or heterogeneous layers, although protective against corrosion, may be less resistant to mechanical wear or fail under cyclic loading, relevant in the areas of dynamic stress in the implants.

From the clinical point of view, changes in Ecorr of the order of 100–200 mV as observed are significant because they indicate a shift of the system towards a more passive regime, reducing metal dissolution in body fluids. Increases in Rp by one or two orders of magnitude can represent drastic reductions in corrosion rates (according to Stern–Geary’s law), resulting in lower metal ion release, most importantly, where corrosion products can induce adverse tissue reactions. Also, this increases implant durability, especially for permanent devices such as orthopedic screws, stents, or dental prostheses. A summary with a comparison between the films is presented in Table 3.

## 4. Conclusions

After thoroughly analyzing the coating samples, the comparison between the data obtained brought us not only to see how the coating effectiveness depends on the substrate angle of inclination (0° vs. 85°), but also to see how it depends on the microarchitecture design. The following conclusions can be obtained regarding the potential implications for the intended biomedical application.

- For conventional geometry (α = 0°), a lower roughness and less developed morphology were observed, with thinner passive layers and lower R_p_ and E_corr_. Thus, in vivo, there is acceptable biocompatibility, but it is potentially inferior to the other geometries due to a lower ability to form stable and protective passive layers. The higher release of metal ions could induce mild inflammatory reactions or less bone integration in implants. It has a better mechanical behavior as it is more homogeneous and has less porous geometry.
- For the inclined geometry (α = 85°), intermediate roughness and diffusion characteristics have been observed, as well as more developed layers than in the conventional one, with improved corrosion resistance (higher R_p_ and R_2_). This implies an improvement in the expected cell integration and osteoblastic adhesion due to the more favorable surface texture, a better passivation that contributes to reducing the release of corrosion products, and a potential compromise between mechanical integrity and biological functionality.
- For zigzag geometry (α = 85°, more complex pattern), higher roughness, surface porosity, and passive multilayer film with better electrochemical behavior have been observed. This implies excellent biocompatibility potential, thanks to lower metal ion release, higher effective surface area generating better interaction with proteins and cells, and possible stimulation of osteogenesis and enhanced cell adhesion. However, mechanical strength and long-term stability should be carefully evaluated, as a highly rough surface may be more prone to wear or delamination.

These results further support the idea that complex surface architectures are to be studied, either by varying the angle of columnar growth or the number of consecutive zigzags, allowing us to obtain surfaces with different properties tailored for specific applications.

## Figures and Tables

**Table 1:** Corrosion parameters of the Ti thin films prepared at normal incidence, inclined, and zigzag geometries in Ringer solution.

Parameter	Conventional (α = 0°)	Inclined (α = 85°)	Zigzag (α = 85°)
E_corr_ (mV vs. Ref.)	−191 ± 12	−116 ± 8	−49 ± 3
I_corr_ (µA)	(7 ± 2.8) × 10^−2^	(1.90 ± 0.8) × 10^−2^	(5 ± 1.6) × 10^−3^
β_c_ (mV/dec)	12.10 ± 2.4	6.40 ± 1.1	15.6 ± 2.7
β_a_ (mV/dec)	11.10 ± 1.5	7.60 ± 0.6	17.1 ± 1.8
Corrosion rate (mpy)	(9 ± 2.4) × 10^−3^	(2.45 ± 0.21) × 10^−3^	(6.44 ± 1.23) × 10^−4^
R_p_ (Ohm)	(2.80 ± 0.21) × 10^4^	(6.4 ± 0.35) × 10^4^	(3.52 ± 0.66) × 10^5^

**Table 2:** Average parameters of the electrical equivalent circuit after fitting the EIS experimental data.

Parameters	Conventional (α = 0°)	Inclined (α = 85°)	Zigzag (α = 85°)
Y1 (Ohm·cm^2^)	1.5 × 10^−4^	1.8 × 10^−4^	1.3 × 10^−7^
n1	0.68	0.52	0.89
R1 (Ohm·cm^2^)	8.6 × 10^3^	1.3 × 10^4^	1.2 × 10^5^
Y2 (S·secn/cm^2^)	7.0 × 10^−5^	9.2 × 10^−10^	6.3 × 10^−8^
n2	0.42	1.00	0.96
R2 (Ohm·cm^2^)	2.8 × 10^3^	3.7 × 10^2^	3.1 × 10^5^
Y3 (S·secn/cm^2^)	1.4 × 10^−6^	2.1 × 10^−6^	9.0 × 10^−5^
n3	0.60	0.69	0.39
R3 (Ohm·cm^2^)	5.7 × 10^3^	1.5 × 10^4^	5.0 × 10^4^
χ^2^	3.7 × 10^−4^	1.3 × 10^−3^	8.5 × 10^−4^

**Table 3:** Summary comparison of experimental results between the films.

Parameter	Conventional (α = 0°)	Inclined (α = 85°)	Zigzag (α = 85°)
Thickness (μm)	1.0 ± 0.1	1.0 ± 0.1	1.0 ± 0.1
Roughness (nm)	8 ± 0.6	32 ± 1.0	30 ± 1.0
Crystallinity	002	101	101
Corrosion rate (mpy)	0.19 ± 0.03	0.04 ± 0.01	0.006 ± 0.0005

## Data Availability

The original contributions presented in this study are included in the article. Further inquiries can be directed to the corresponding author.
